# Significant Disparity of Access to Stroke Treatment Between the Western Parts and Eastern and Northern Parts of Sydney

**DOI:** 10.7759/cureus.44285

**Published:** 2023-08-28

**Authors:** Behzad Eftekhar

**Affiliations:** 1 Neurological Surgery, University of Sydney, Sydney, AUS; 2 Neurological Surgery, Macquarie University, Sydney, AUS

**Keywords:** healthcare equity, healthcare disparity, health advocacy, clot retrieval, simulation research, computer simulation, stroke systems of care, health policy and advocacy

## Abstract

Objective

To provide an estimate of access times and distances to an endovascular clot retrieval (ECR) service provider for a typical stroke patient in the western part of Sydney and to compare it with the eastern and northern parts.

Methods

Incidences of stroke were simulated through a population-weighted randomized selection of addresses in the studied western, eastern, and northern areas of Sydney (100,000 times for each). The access times and distances were calculated from those addresses to the closest ECR hub for the eastern and northern parts and to all five ECR hubs, as well as the Nepean Public Hospital (NPH) for the western part. The access times and distance means were compared statistically using ANOVA.

Results

In the western areas, the estimated average access times and distances to different ECR hubs varied from 38.5 (+/- 15) to 45 (+/- 15) minutes and from 42 (+/- 15.9) to 46.8 (+/- 16) km in working hours and from 45 (+/- 15) to 64 (+/- 15) minutes and 46.8 (+/- 16) to 69.6 (+/- 16) km in after hours. However, the estimated average access times and distances to the local ECR hub were 12.25 (+/- 6) minutes and 9.1 (+/- 5.6) km for northern and 7.5 (+/- 4) minutes and 4.4 (+/- 2.5) km for the eastern areas. The differences between the estimated average access times and distances for a typical stroke patient to an ECR hub in the western areas in comparison with eastern or northern areas were statistically significant (p<0.0001). The average access times and distances in the western part to NPH were 17 (+/- 16) minutes and 15.6 (+/- 16.6) km.

Conclusions

The patients in the western part of Sydney had significantly longer access times to ECR hubs than those living in comparable areas of the eastern and northern parts. This study supports the Nepean Public Hospital supplying an ECR service to achieve travel times, and, therefore, treatment times for a typical stroke patient in the western parts, similar to patients in the eastern and northern parts of Sydney.

## Introduction

Time has become even more crucial in the treatment of embolic brain strokes [1,2], with the advent of endovascular clot retrieval (ECR) [3-7]. According to Saver [8], the typical patient loses 1.9 million neurons, 14 billion synapses, and 12 km (7.5 miles) of myelinated fibers each minute in which stroke is untreated. In a recent meta-analysis of pooled individual patient data from 406 adults in seven randomized trials, in the time interval from hospital arrival to endovascular procedure start, every 1 s of delay was associated with a loss of 2.2 hours of healthy life [9]. Optimizing access to ECR treatment for all patients at risk of stroke shall be a priority in public health policy and treatment planning for stroke patients.

There are seven public hospitals in New South Wales (Royal Prince Alfred (RPA), Prince of Wales (POW), Royal North Shore (RNS), John Hunter Hospital, Liverpool hospital (LPH), Westmead hospital (WMH), and Nepean Public Hospital (NPH)) that have equipment and qualified specialists to provide ECR service; however, only the first six are permitted to provide ECR service.

Currently, stroke patients in the catchment area of NPH in the western part of Sydney are transferred to WMH or LPH during working hours for ECR and to RNS hospital, LPH, POW, or RPA hospital after hours. Depending on the circumstances, transfer to RPA, RNS, or POW may be considered at any time.

The purpose of this study was to investigate the estimate of access times to an ECR service provider for a typical stroke patient in the western part of Sydney under current triage protocols and compare it with the access time estimates in comparable areas in the eastern (catchment area of POW) and northern (catchment area of RNS) parts of Sydney. As a typical stroke patient in the western parts of Sydney could potentially be transferred to WMH, LPH, RNS, RPA, or POW for ECR, the access times to all these hospitals were included in the study.

## Materials and methods

Data

In order to calculate and compare the estimate of access times and distances, we needed to select comparable areas in the western, eastern, and northern parts of Sydney.

Geographical information and estimated resident populations of 18 statistical areas (SA) level 2 of two western SA3 areas (ASGS2021) (Penrith - SA3 code 12403; Blue Mountains - combined SA3 codes 12401 and 12402), 12 SA2 areas of two north Sydney SA3 areas (Ku-ring-gai - SA3 code 12103; north Sydney - SA3 code 12104) and 18 SA2 areas of two eastern Sydney SA3 areas (eastern suburbs - north - SA3 code 11801 - and eastern suburbs - south SA3 code 11802) were obtained from the Australian Bureau of Statistics (ABS) website [10]. The studied eastern, western, and northern SA3 areas are referred to as east, west, and north in the text, respectively. 

The selected areas were all within driving distance of an ECR-capable hospital, and their populations were comparable (220,351 for the studied northern areas; 222,494 for the western areas; and 290,338 for the eastern areas according to the ABS regional population report (released on 26/07/2022; www.abs.gov.au).

**Figure 1:**
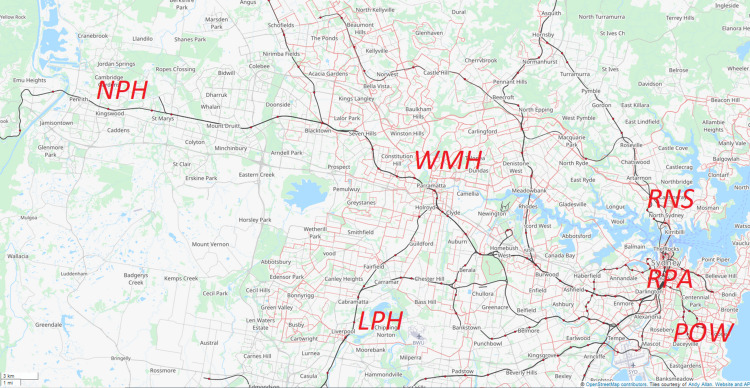
Locations of Royal Prince Alfred (RPA), Prince of Wales (POW), Royal North Shore (RNS), Liverpool hospital (LPH), Westmead hospital (WMH), and Nepean Public Hospital (NPH). The figure was created using OpenStreetMap.org. The data are available under the Open Database License [11].

The geographical coordinates (longitude and latitude) of SA2 areas of the abovementioned SA3 areas (one for each SA2 area) and also RNS hospital, LPH, RPA hospital, POW hospital, WMH, and NPH were extracted using both Google Maps and OpenStreetMap.org online facilities. Figure 1 shows the location of these hospitals.

Computational simulation model

Basically, we simulated incidences of stroke in the eastern, northern, and western parts of Sydney using computer-generated random addresses, calculated their distances from ECR hubs, and compared them statistically.

As incidences of stroke are expected to be higher in more populated areas, using the above-mentioned extracted coordinates, three separate lists of coordinates (one for the west, one for the north, and one for the east) were generated in which each pair of longitude and latitude was repeated proportionately with regards to the estimated population of each related SA2 area. In order to minimize chance-related errors of the calculations, 100,000 pairs of coordinates were randomly picked by computer using Python (3.10) codes from each one of the three lists. Each randomly selected pair of coordinates simulated the potential location of an incidence of stroke. In the east and north, the access distance to the ECR hub for a typical stroke patient during working hours (8 am to 5 pm, Monday to Friday) is not different from after hours (out of working hours), because the north and east each have a dedicated ECR hub (RNS for the north and POW for the east). However, in the west, the travel times and distances for a typical stroke patient during working hours are different from that after hours, because, in working hours, the patients can be transferred to WMH as well as LPH, while, after hours, they are transferred to hospitals other than WMH, including LPH, RNS, RPA, or POW. Based on this, travel time and distance (by car) of each pair of coordinates in the north from RNS, in the east from POW, and in the west from each of six hospitals were calculated on 19/02/2023, using the Open Source Routing Machine (version 5.14) module in STATA (StataCorp. 2021. Stata Statistical Software: Release 17. College Station, TX: StataCorp LLC) [12].

STATA MP 17 (StataCorp. 2021. Stata Statistical Software: Release 17. College Station, TX: StataCorp LLC) was used for descriptive analysis and one-way ANOVA with post-hoc comparisons using Bonferroni multiple-comparison tests, comparing travel times and distances for a typical stroke patient to get to an ECR hub in the west (during working hours from randomly generated addresses in the west to LPH or WMH), east, and north.

## Results

This study shows that the estimated average travel time and distance to ECR hubs for stroke patients in the western Sydney areas were significantly longer than for the stroke incidences in the northern and western Sydney areas. In the western areas, the travel time was 38.47 (+/- 15) to 45 (+/- 15) minutes in working hours and 45 (+/- 15) to 64 (+/- 15) minutes in after hours versus 12.25 (+/- 6) minutes for the northern and 7.5 (+/- 4) minutes for the eastern areas. The travel time for patients in the western areas is longer after hours because WMH (ECR hub closer to the west ) is taken out of calculations. The estimated average travel distance for stroke patients in the western Sydney areas was 42 (+/- 15.9) to 46.8 (+/- 16) km in working hours and 46.8 (+/- 16) to 69.6 (+/- 16) km in after hours versus 9.1 (+/- 5.6) km for northern and 4.4 (+/- 2.5) km for eastern Sydney areas. The travel time and distance for a typical patient with stroke in the western Sydney areas to access NPH were 17 (+/- 16) minutes and 15.6 (+/- 16.6) km on average (Tables 1-2). The differences between areas were statistically significant (Tables 3-4; for time: F=3.9e+05, between groups df=2, p<0.0001; for distance: F=5.1e+05, between groups df=2, p<0.0001). Post-hoc comparisons using the Bonferroni multiple-comparison test confirmed that the mean travel time and distance to an ECR hub for a typical stroke patient in the west (during working hours) is significantly longer than those in the east (p<0.0001 for time and p<0.0001 for distance) or north (p<0.0001 for time and p<0.0001 for distance).

**Table 1:** Travel time for a typical stroke patient in the studied areas to get to an ECR hub

Travel time for a typical stroke patient to get to an ECR hub	Obs (Randomly selected addresses by the computer)	Mean (seconds)	Std. dev.	Min (seconds)	Max (seconds)
In the east	100,000	450.93 (7.5 minutes)	216.72	111.3	814.6
In the north	100,000	735.17 (12.25 minutes)	365.09	167.4	1,256.4
In the west if the patient goes to Prince of Wales	100,000	3,839.62 (64 minutes)	907.05	2,944.1	9,923.9
In the west if the patient goes to Royal North Shore	100,000	3,648.45 (60.8 minutes)	939.43	2,824.5	9,125.5
In the west if the patient goes to Royal Prince Alfred	100,000	3,354.11 (55.9 minutes)	909.05	2,455.1	9,556
In the west if the patient goes to Liverpool	100,000	2,685.52 (45 minutes)	904.80	1,806.8	9,088.1
In the west if the patient goes to Westmead	100,000	2,308.31 (38.47 minutes)	908.98	1,409.3	8,315.7
In the west if the patient goes to Nepean	100,000	1,022.1 (17 minutes)	972.3	284	7,806.1

**Table 2:** Travel distance for a typical stroke patient in the studied areas to get to an ECR hub

Travel distance for a typical stroke patient to get to an ECR hub	Obs (Randomly selected addresses by the computer)	Mean (meters)	Std. dev.	Min (meters)	Max (meters)
In the east	100,000	4,432.90 (4.4 kilometers)	2,488.49	783.3	1,0704.1
In the north	100,000	9,130.58 (9.1 kilometers)	5,649.82	1,410.4	17,395.4
In the west if the patient goes to Prince of Wales	100,000	69,685.38 (69.6 Kilometers)	15,930.6	52,534.7	166,400.4
In the west if the patient goes to Royal North Shore	100,000	65,626.43 (65.6 kilometers)	16,616.54	50,557.5	153,540.9
In the west if the patient goes to Royal Prince Alfred	100,000	61,260.76 (61.2 kilometers)	15,942.83	44,054.5	156,013.2
In the west if the patient goes to Liverpool	100,000	46,771.57 (46.8 kilometers)	16,028.8	29,986.9	148,771.3
In the west if the patient goes to Westmead	100,000	41,987.1 (42 kilometers)	15,942.1	2,4781	134,223.9
In the west if the patient goes to Nepean	100,000	15,628.79 (15.6 kilometers)	16,628.74	2,823.5	125,888.2

**Table 3:** Results of oneway ANOVA comparing travel times for a typical stroke patient to get to an ECR hub in the west, east, and north Bartlett's equal-variances test: chi2(2) = 2.4e+05, Prob>chi2 = 0.000

Travel time for a typical stroke patient to get to an ECR hub	Mean (seconds)	Std. dev.	Freq		SS	df	MS	F	Prob > F
In the east	450.93	216.72	100,000	Between groups	3.6651e+11	2	1.8325e+11	3.9e+05	0.0000
In the west	2,496.91	926.30	200,000	Within groups	1.8963e+11	399,997	474,083.034		
In the north	735.17	365.09	100,000						

**Table 4:** Results of one-way ANOVA comparing travel distances for a typical stroke patient to get to an ECR hub in the west, east, and north Bartlett's equal-variances test: chi2(2) = 3.4e+05, Prob>chi2 = 0.000

Travel distance for a typical stroke patient to get to an ECR hub	Mean (Meters)	Std. dev.	Freq		SS	df	MS	F	Prob > F
In the east	4,432.90	2,488.49	100,000	Between groups	1.4246e+14	2	7.1231e+13	5.1e+05	0.0000
In the west	4,4379.34	16,163.48	200,000	Within groups	5.6063e+13	399,997	140,157,616		
In the north	9,130.58	5,649.82	100,000						

## Discussion

This study not only shows that there is a significant discrepancy in access to stroke treatment in the western parts compared with the eastern and northern parts of Sydney but also shows that the travel time for a typical patient with stroke in western Sydney areas to access ECR could potentially be reduced from 38-64 minutes to 17 minutes on average if Nepean hospital is permitted to provide ECR services.

The computational simulation model has been based on selected areas in western, northern, and eastern parts of Sydney that are within driving distance to an ECR-capable public hospital with comparable populations. The selected areas could be considered representatives of the western, northern, and eastern parts of Sydney.

There are no similar published studies to compare our results with. Phan et al., in their studies [13,14] about utilizing Google service boundaries for ECR hub hospitals in a metropolitan setting, used a different approach to calculate the access times to ECR hubs. They generated random coordinates in each suburb of metropolitan Melbourne and converted them to addresses and their governing postcodes using reverse geocoding. Reverse geocoding was used to check that the randomly generated coordinates lay within a postcode, and if not, the relevant coordinate was removed, and another random coordinate was generated in its place. This step was repeated until the estimated number of stroke cases in that postcode based on published stroke incidence data in 2000 [15] has been reached. The method in this study is not dependent on the stroke incidence data and has fewer chance-related errors due to a high number of travel time and distance calculations. However, it is assumed that the number of strokes is proportionate to the population.

While using online mapping services (e.g., Google API) may provide more accurate and real-time travel distance and time calculations, the calculation method used in the current study has some advantages. The method used in this study is independent of the Internet and commercial providers, and as it is an offline procedure that uses only open-source software, it ensures that the results can be replicated at any time and carries no risk of the computer code becoming obsolete [16,17]. Although a real-time calculation is sometimes desired, it is more important, particularly for the purpose of health care planning, to be able to access results that can be replicated at any time, which is not possible when using real-time data from online services as the results are a function of time-specific circumstances.

From a financial perspective, the treatment cost per stroke patient does not change if a hospital that already has the equipment and specialist manpower (NPH) is permitted to provide ECR service. In other words, the consumable medical expenses per patient remain the same irrespective of the location of the ECR procedure. There are expected savings on paramedic resources considering the reduction in the patients’ transfer requirements between hospitals. The shorter the time to treatment for stroke, the better the patient’s outcome will be and the less the long-term expenses of the stroke patients’ rehabilitation [18]. In a recently published meta-analysis, every 1 s delay was associated with a loss of 2.2 hours of healthy life [9]. This estimates on average potential prevention of loss of 2772-6204 hours (115.5-258.5 days) of healthy life for each local stroke patient if NPH provides ECR service. Further comparative study of the stroke outcomes based on the patients’ addresses and ECR hubs is required.

Provision of service for stroke patients by local hospitals can help the community as they may need to travel shorter distances to the hospital and back during the treatment period.

Limitations of the study

The calculation method has not taken into consideration the peak hours traffic delays, which add to the travel time and distance during peak hours. According to the Australian Bureau of Infrastructure, Transport and Regional Economics (BITRE) Information Sheet published in 2015, urban trips (without specifying location) performed throughout the day, which would take an hour during uncongested times, take on average about 20 minutes longer due to traffic delays [19].

Ambulances may have different travel times than other vehicles because they are able to operate with lights and sirens if necessary. The estimates of travel time were calculated using Open Source Routing Machine, so the actual travel time may be different from the estimates for ambulances in different circumstances. However, considering the extent of the differences, it would not change the main conclusion and principal message.

No formal financial analysis or costings has been done to support the comments about the treatment cost per stroke patient if a hospital that already has the equipment and specialist manpower (NPH) is permitted to provide ECR services.

## Conclusions

The study found that patients in the western part of Sydney had significantly longer access times to ECR treatment than those in the northern and eastern parts. As NPH already has the equipment and qualified manpower for ECR services, this study supports NPH supplying ECR services to achieve travel times and, therefore, treatment times for a typical stroke patient in the western parts, similar to patients in the eastern and northern parts of Sydney.
